# A Comprehensive Study on the Occurrence of Mycotoxins and Their Producing Fungi during the Maize Production Cycle in Spain

**DOI:** 10.3390/microorganisms8010141

**Published:** 2020-01-20

**Authors:** Marta García-Díaz, Jéssica Gil-Serna, Covadonga Vázquez, María Nieves Botia, Belén Patiño

**Affiliations:** 1 Department of Genetics, Physiology and Microbiology, Faculty of Biology, University Complutense of Madrid, Jose Antonio Novais 12, 28040 Madrid, Spain; martga43@ucm.es (M.G.-D.); covi@ucm.es (C.V.); belenp@ucm.es (B.P.); 2 Laboratorio Arbitral Agroalimentario, Aguaron 13, 28023 Madrid, Spain; nbotia@tragsa.es

**Keywords:** aflatoxins, fumonisins, good agricultural practices, corn, PCR-detection, *Aspergillus*, *Fusarium*

## Abstract

Mycotoxin contamination is one of the main problems affecting corn production, due to its significant risk to human and animal health. The *Fusarium* and *Aspergillus* species are the main producers of mycotoxins in maize, infecting both pre-harvest and during storage. In this work, we evaluated the presence of mycotoxins and their producing species along maize production cycles in three different stages (anthesis, harvest, and storage) during three consecutive seasons (2016–2018). Fungal occurrences were studied using species-specific PCR protocols, whereas mycotoxin levels were determined by LC-MS/MS. Fumonisin-producing *Fusarium* species (*F. verticillioides* and *F. proliferatum*), as well as the aflatoxin producer *Aspergillus flavus*, were the most predominant species at all stages; although, during some seasons, the presence of *F. graminearum* and *A. niger* aggregate species were also identified. Contrastingly, fumonisins were the only mycotoxins detected and levels were always under legal regulations. The results presented here demonstrate that even when fungal contamination occurs at the early stages of the maize production cycle, the application of good agricultural and storage practices might be crucial to ensure mycotoxin-free grains.

## 1. Introduction

Maize (*Zea mays* L.) is a monoecious plant of the Poaceae family, and it is cultivated worldwide. Two phases can be distinguished during its growth—vegetative and reproductive. The vegetative phase begins with the growth of the plant and culminates with the appearance of the male flower. The reproductive phase begins with the emergence of the female flower, and the physiological maturity of the cob takes place in this stage [1]. Maize is one of the most important cereals, with an annual worldwide production of 1134 million tons in 2017, according to the Food and Agriculture Organization of the United Nations [2].

One of the main problems regarding corn production is mycotoxin contamination. Mycotoxins are toxic secondary metabolites, produced by several fungi, that frequently contaminate maize in the field and/or during storage [3]. The most relevant fungal genera affecting maize are *Aspergillus* and *Fusarium* [4]. The main mycotoxins associated with corn during all of its production cycles and its storage are fumonisins (FUMs), trichothecenes (TCTs), zearalenone (ZEA), aflatoxins (AFs), and ochratoxin A (OTA) [5].

Maize grains are often contaminated simultaneously with various mycotoxin-producing species, the most relevant being *Fusarium verticillioides* and *F. proliferatum*, the main FUM-producing species; *F. graminearum*, which produces TCTs and ZEA; and *Aspergillus flavus*, the main AF-producing species [6]. 

The consumption of corn contaminated by mycotoxins may cause a number of severe toxic effects in both animals and humans. The International Agency for Research on Cancer (IARC) has classified AFB_1_ as a carcinogen to humans (group 1) and fumonisin B_1_ (FB_1_) and OTA as possible human carcinogens (group 2B) [7]. Due to the health risk associated with the intake of foodstuffs contaminated by mycotoxins, the European Union has established—through legislation—the maximum levels of certain toxins allowed in maize and its derived products. The European Commission (CE) Regulation N° 1881/2006 [8] set the maximum levels for AFs, FUMs, ZEA, and Deoxynivalenol (type B TCT) in unprocessed maize and different maize-derived products intended for human consumption. This regulation was subsequently modified by CE N° 1126/2007 [9] and CE N° 165/2010 [10] to apply more restrictions on the level of AFs and *Fusarium* toxins allowed in these products. Currently, there is no legislation setting the maximum levels of T-2 and HT-2 (type A TCTs) or OTA in corn, due to a lack of studies regarding their relevance in this food matrix.

The presence of these toxins, apart from constituting a threat to human and animal health, may cause serious economic losses to farmers, who have to discard contaminated grains [11]. Moreover, feed contamination by mycotoxins also increases veterinary care costs and severely reduces livestock production [11].

Traditionally, the species included in the *Fusarium* genus are considered field fungi, although FUM production often happens during post-harvest, when corn storage conditions are inadequate and permissive for toxin production [12]. On the other hand, *A. flavus*—which has been traditionally considered a storage fungus—can infect maize both pre-harvest and during storage, and an increase in AF content is likely if the drying and storage conditions are not appropriate [6]. However, there are not currently enough data to support this statement. Therefore, it is essential to know what happens during the maize production cycle and to establish adequate control methods to avoid mycotoxins entering the food chain.

Mycotoxins are introduced into food chains by the pre- or post-harvest contamination of food and feed [13]. The early detection of toxin-producing species contaminating maize is one of the most important actions in preventing mycotoxin contamination [14]. The identification of these fungal species has been traditionally performed using traditional isolation and culturing techniques [15]. However, these conventional methods for the identification and detection of these fungi in food and feed are time consuming and require taxonomical expertise [16]. Hence, it is important to develop fast and reliable techniques to detect mycotoxin producers within foodstuffs. PCR-based protocols that target DNA are considered a good alternative for rapid diagnosis, due to their high specificity and sensitivity and have been used for the detection of toxigenic species in maize and maize products [17]. The main advantages of species-specific PCR protocols are that they offer a sensitive, high-throughput method for identifying fungi in complex mixtures, even when they are no longer viable [18]. It is not necessary to culture the organisms for a long time, only 24–48 h before detection—which significantly reduces the time of the analysis [15,16].

The aim of this study is to evaluate, for the first time, the presence of mycotoxin-producing species along maize production cycles at three different stages (anthesis, pre-harvest, and storage) and its possible relationship with mycotoxin contamination. For this purpose, we analyze the samples with previously optimized PCR protocols or new ones developed in this work. Subsequently, mycotoxin levels are detected by LC/MS/MS.

## 2. Materials and Methods

### 2.1. Corn Samples and Fungal Isolates

#### 2.1.1. Corn Samples

Maize samples of different varieties (DKC 6630, DKC 6442, and DKA 6728) were collected from a farm located in the South Area of the Community of Madrid (Spain), at three crop stages (anthesis, pre-harvest, and storage), along three consecutive seasons (2016, 2017, and 2018). In this farm, good agricultural practices (GAP) and adequate fallow periods are correctly applied. The study was performed sampling different continuous plots (P); two plots were sampled in 2016 (P1-P2) whereas three different plots were evaluated in 2017 (P1-P2-P3) and 2018 (P2-P3-P4). A total of 27 samples were evaluated (Table 1).

The sampling procedure for each plot was set as follows: (1) in the anthesis period, 25 male flowers (MF) and 25 female flowers (FF) were collected; (2) during pre-harvest (approximately 7 days before harvest), 25 cobs (PRE) (grain moisture 35%) were sampled; (3) after three months of storage, 3 kg of corn grain from different points of the silo were collected. The relative humidity and temperature were registered every 8 h during silo storage in 2018 using a data logger El-USB-1 (LASCAR electronic, Salisbury, UK).

For each plot, the samples were collected at random, taking one sample every 3 m along the plot. When they arrived at the laboratory, all the samples were separated into three lots. Subsequently, flower samples, cobs of pre-harvest (previously threshed), and silo samples were crushed with an IKA A11 Basic Mill (IKA, Königswinter, Germany) to obtain a fine powder, according to European Union requirements [19]. The milled samples were placed in hermetic sterile plastic bags and stored at −20 °C until analysis. All samples were analyzed in triplicate.

#### 2.1.2. Fungal Isolates and Culture Conditions

All the isolates used in this study to optimize species-specific PCR protocols—along with their sources—are listed in Table 2. The fungal strains came from different culture collections or they were isolated in our laboratory from Spanish cereal samples.

The strains were maintained by regular sub-culturing on potato dextrose agar medium (PDA) (Pronadisa, Madrid, Spain) at 25 ± 1 °C for 5 days and stored as spore suspensions in 15% glycerol (Panreac, Madrid, Spain) at −80 °C until required.

### 2.2. Primer Design and PCR Amplification

Specific primer sets were designed on the basis of sequence alignments of the partial region of the elongation factor 1α (*tef-1α*) encoding gene. For the alignments, sequences of more than twenty strains from different origins were used. The alignments included sequences from the *Fusarium* species, as well as from other related species obtained in this paper, or in previous works carried out in our laboratory, or retrieved from the NCBI database. The sequences were edited and aligned using the ClustalW method, using UGENE 1.29 software (Unipro, Novosibirsk, Russia).

Genomic DNA extraction from *Fusarium* strains listed in Table 2 was carried out using the protocol described elsewhere [20]. Fungal mycelia from four-day-old cultures on PDA plates were scraped off with a scalpel, frozen with liquid nitrogen and ground using a micropistille prior to DNA extraction. DNA concentrations were determined using a NanoDrop^®^ ND-1000 spectrophotometer (Nanodrop Technologies, Wilmington, NC, USA). All PCR assays were performed in an Eppendorf Mastercycler Gradient (Eppendorf, Hamburg, Germany). Genomic DNAs were amplified using primers EF-1 and EF-2 [21], which amplify a partial region of the *tef-1α* gene. The amplification reactions were carried out in volumes of 25 μL, containing 100 ng of sample DNA, 1 μL of each primer (20 μM; Metabion, Planegg, Germany) and 12.5 μL of NZYTaq II 2x Green Master Mix (Nzytech, Lisbon, Portugal). 

PCR products were detected in 2% agarose ethidium bromide gels in TAE 1X buffer (Tris Acetate 40 mM and EDTA 1.0 mM). The NZYDNA Ladder V (Nzytech, Lisbon, Portugal) was used as the molecular size marker.

The amplification products approximately 670 bp-long were purified using the NZYGelpure Kit (Nzytech, Lisbon, Portugal) and sequenced with an ABI PRISM 3730XL DNA sequencer (Applied Biosystems, Foster City, CA, USA), according to manufacturer’s instructions in Macrogen facilities (Madrid, Spain). All amplification products were sequenced in both directions. 

The sequences were assembled using the UGENE 1.29 package. The sequences were compared with those deposited on NCBI nucleotide databases to reach the identification of the corresponding isolates at species level. Subsequently, these sequences were deposited into the NCBI database.

Specific PCR assay of *F. graminearum* was carried out using primers GRAM.EF-F (5′-AACCCCGCCGACACTTGGCG-3′) and GRAM.EF-R (5′-GGTTGACACGTGATGATGAGCG-3′), and the following protocol: 1 cycle of 5 min at 94 °C, 30 cycles of 35 s at 95 °C (denaturation), 45 s at 66 °C (annealing), 30 s at 72 °C (extension) and, finally, 1 cycle of 5 min at 72 °C, was followed.

In the case of *F. langsethiae*, the primers were LANG.EF-F (5′-GCTCTTCCTTCCCACATAGCCA- 3′) and LANG.EF-R (5′-GCAGGCATGTTAGTATGATAATG-3′) and the protocol was: 1 cycle of 5 min at 94 °C, 28 cycles of 35 s at 95 °C, 20 s at 62 °C, 30 s at 72 °C and, finally, 1 cycle of 5 min at 72 °C. 

For *F. fujikuroi*-specific detection, the primers designed were FUJI.EF.F (5′-TTGCCCACCGATTTCCCTTACGAT-3′) and FUJI.EF.R (5′-GTTAGTATGAATAAGTAGAATGAAGCAT-3′), and the protocol was: 1 cycle of 5 min at 95 °C, 30 cycles of 35 s at 95 °C, 30 s at 61 °C, 30 s at 72 °C and, finally, 1 cycle of 5 min at 72 °C. 

The primer set POAE.EF-F (5′-GCATTTCTTTGGGCGCGAATCG-3′) and POAE.EF-R (5′TGAGTGACTGAGGTAGTAGTGAC 3′) was used in the case of *F. poae*, using the protocol: 1 cycle of 5 min at 94 °C, 30 cycles of 35 s at 95 °C, 20 s at 66 °C, 30 s at 72 °C and, finally, 1 cycle of 5 min at 72 °C. 

A specific PCR assay of *F. sporotrichioides* was performed using the primers SPORO.EF-F (5′-GCTTTTGCCCTTCCCACACAT-3′) and SPORO.EF-R (5′-AATGTGATGAAGGCAATAGTGAC-3′), and the protocol: 1 cycle of 5 min at 94 °C, 30 cycles of 35 s at 95 °C, 20 s at 62 °C, 30 s at 72 °C and, finally, 1 cycle of 5 min at 72 °C. 

Finally, in the case of *F. temperatum* the primers designed were TEMP.EF-F (5′-CAAGACCTGGCGGGCATCTCA-3′) and TEMP.EF-R (5′-CTCAGAAGGTTGTGGCAATGG-3′) and the protocol: 1 cycle of 5 min at 95 °C, 27 cycles of 30 s at 95 °C, 30 s at 64 °C, 25 s at 72 °C and finally 1 cycle of 5 min at 72 °C. 

### 2.3. Study on the Occurrence of Mycotoxins and Mycotoxin-Producing Fungi on Maize Samples

#### 2.3.1. PCR Detection of the Main Mycotoxin-Producing Fusarium and Aspergillus Species

##### DNA Extraction

Before DNA extraction, 1 g of the milled sample was cultured in 250 mL Erlenmeyer flasks containing 50 mL Sabouraud–Chloramphenicol broth (Pronadisa, Madrid, Spain), at 28 °C on an orbital shaker SK-0330-PRO (140 rpm) (Labolan, Navarra, Spain) in darkness for 24 h. Subsequently, the cultures were filtered through Whatman N° 1 paper. Filtered cultures were frozen with liquid nitrogen and ground using a mortar and pestle. The samples were kept at −80 °C until DNA extraction.

For each sample, DNA extraction was carried out in triplicate using the NZYPlant/Fungi gDNA Isolation Kit (Nzytech, Lisbon, Portugal) according to the manufacturer’s instructions (DNA extraction protocol from fungi). DNA concentrations were determined using a NanoDrop^®^ ND-1000 spectrophotometer (Nanodrop Technologies, Wilmington, NC, USA).

##### Detection Aspergillus and Fusarium Species, by Specific PCR Assays

Species-specific PCR protocols were applied to detect the aflatoxin (AF) producers *A. flavus* [22] and *A. parasiticus* [23]. The presence of the most relevant ochratoxin A (OTA) producers (*Aspergillus carbonarius*, *A. westerdijkiae*, *A. ochraceus* and *A. steynii*) was also evaluated using protocols previously described in our laboratory [24,25]. Some *A. niger* aggregate species (*A. niger* and *A. welwitschiae*) are able to produce both OTA and fumonisin B_2_ (FB_2_), and their presence was also tested using the specific protocols described by Palumbo [26]. 

The presence of the fumonisin-producing *Fusarium* species was also tested using species-specific PCR protocols. Some assays are described in the present work (*F. temperatum* and *F. fujikuroi*), whereas other protocols were previously described by our group for their use in detecting *F. proliferatum* [27] and *F. verticillioides* [28]. The specific primer pair for *F. subglutinans* was designed by Scauflaire et al. (2012) [29], although it was used with the amplification protocol described for *F. temperatum* (Section 2.2). PCR protocols were also applied to detect the trichothecenes (TCT)- and zearalenone (ZEA)-producing *Fusarium* species. *F. graminearum* was evaluated using the protocol described here, whereas *F. equiseti* and *F. culmorum* were detected by the protocol described in Jurado et al. (2005) [17]. The specific detection of the type A TCT-producing species *F. poae*, *F. sporotrichioides* and *F. langsethiae* was performed using the protocols described in this article. 

The PCR assays and the detection of the PCR products were carried out as described in the Section 2.2 (primer design and PCR amplification). Before the test using specific protocols, the presence of fungal DNA in all samples was confirmed using universal primers ITS1/ITS4 [30].

#### 2.3.2. Mycotoxin Determination

Mycotoxin analyses were performed in the “Laboratorio Arbitral Agroalimentario” (Madrid, Spain) following its standardized protocols. The presence of AFB_1_, OTA, fumonisins B_1_ and B_2_ (FB_1_ and FB_2_), TCT type A (T-2 and HT-2 toxins) and type B (deoxynivalenol (DON)) and ZEA were analyzed in the pre-harvest and stored maize samples.

To determine the toxin concentrations of the samples, 5 g of dry milled maize was thoroughly mixed with 25 mL acetonitrile/water/formic acid (79:20:1, *v*/*v*) in polypropylene tubes. The mixture was shaken in an orbital shaker (Excella^®^ E24, New Brunswick Scientific, Eppendorf, Germany) for 30 min to extract toxins, and then centrifuged at 5000 rpm for 5 min. Subsequently, 500 μL of extract were collected in a vial, then 25 μL of internal standards solution was added. The solvent was evaporated in a gentle stream of N_2_ at 50 °C, and the residue was solved in 250 μL of mobile phase B, then 250 μL of mobile phase A was added. The extracts were filtered using cellulose syringe filter, with a 0.22 μm pore size (Minisart^®^, Sartorius Stedim, Germany) and then were transferred into vials and stored at −20 °C until analysis. All samples were performed by duplicate.

The samples were examined by LC-MS/MS using a 325 LC/MS system (Varian Inc., Palo Alto, CA, USA), equipped with an ESI interface and HPLC system, with a 212 LC binary pump and a 460 LC automatic microinjector from Agilent Technologies (Waldbronn, Germany). 

Separation was performed on an EC-C18 column (Poroshell 120 Agilent 50 × 4.6 mm, 2.7 μm particle size). The column temperature was 25 °C. The injection volume was 20 μL. The mobile phase consisted of a mixture of 0.15% formic acid with 0.5 mM ammonium formate in water (A) and 0.1% formic acid in methanol (B) and the gradient was programmed as follows: 0.00 min; 90% A, 10% B. 1.42 min; 90% A, 10% B. 2.54 min; 60% A, 40% B. 15.06 min; 35% A, 65% B. 15.30 min; 0% A, 100% B. 22.00 min; 0% A, 100% B. 22.54 min; 90% A, 10% B. 30.00 min; 90% A, 10% B. The flow-rate was 250 μL/min.

Standard curves were constructed with different levels of mycotoxin standards (Biopure™, Romerlabs, Tulln an der Donau, Austria). The detection limits were of 2 μg/kg in the case of AFB_1_, 6 μg/kg for OTA, 180 μg/kg for FB_1_, 60 μg/kg for FB_2_, 6 μg/kg for T-2, 8 μg/kg for HT-2, 80 μg/kg for DON, and 12 μg/kg for ZEA.

## 3. Results

### 3.1. Optimization of Species-Specific PCR Protocols

The sequences of the partial region of the *tef-1α* gene were obtained from several *Fusarium* strains and deposited on the NCBI database with accession numbers between MN861741 and MN861808. Some *tef-1α* sequences already available on databases were also included in the alignments. On the basis of these alignments, species-specific primers and PCR protocols were designed in order to detect *Fusarium graminearum*, *F. langsethiae*, *F. fujikuroi*, *F. poae, F. sporotrichioides* and *F. temperatum.* The specificity of the PCR assays developed was tested using DNA isolated from a wide range of fungal strains (Table 2). 

**Figure 1:**
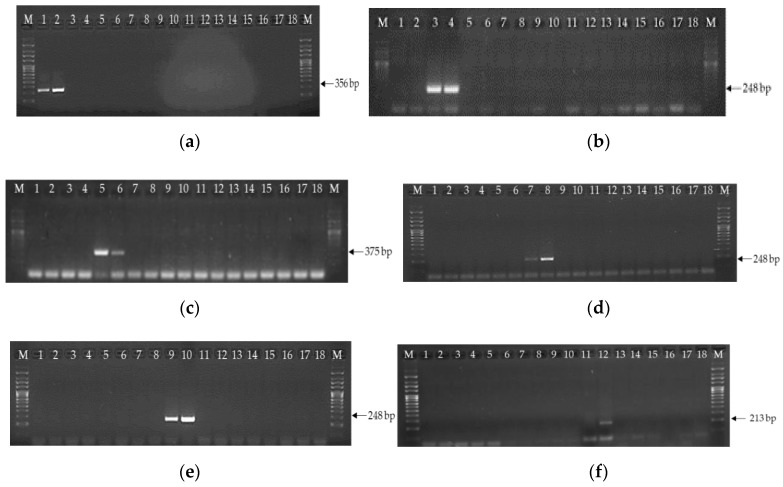
PCR amplification using the optimized protocols for specific detection of *F. graminearum* (**a**) *F. langshetiae* (**b**), *F. fujikuroi* (**c**) *F. poae* (**d**), *F. sporotrichioides* (**e**) and *F. temperatum* (**f**). Lanes 1 and 2: *F. graminearum* NRRL 28585, NRRL 28436; Lanes 3 and 4: *F. langsethiae* L.3.1, L.3.2*;* Lanes 5 and 6: *F. fujikuroi* ITEM 4092, ITEM 4093; Lanes 7 and 8: *F. poae* ITEM 6006, ITEM 6007; Lanes 9 and 10: *F. sporotrichioides* ITEM 550, ITEM 695; Lane 11: *F. subglutinans* CBS 747.97; Lane 12: *F. temperatum* CBS 138.287; Lane 13: *F. culmorum* ITEM 628; Lane 14: *F. globosum* ITEM 613; Lane 15: *F. equiseti* VIo1093; Lane 16: *F. verticillioides* F1-VERT; Lane 17: *F. proliferatum* F2-PRO; Lane 18: non template control. M: DNA molecular size 1000 bp marker.

As an example, Figure 1 shows the agarose gel electrophoreses and the results after the application of the species-specific PCR protocols designed using DNA from relevant *Fusarium* species. Figure 1a shows the results using GRAM.EF-F/GRAM.EF-R primers and their specific protocol, which amplified a single fragment of 356 bp solely when the genomic DNA of *F. graminearum* strains was used. Figure 1b,d, and e show the results of specific assays which used LANG.EF-F/LANG.EF-R, POAE.EF-F/POAE.EF-R and SPOR.EF-F/SPOR.EF-R primer sets, respectively, which amplified a single fragment of 248 bp, when genomic DNAs of *F. langsethiae, F. poae* or *F. sporotrichioides* strains were used. Similarly, the PCR amplifications of genomic DNA from all the isolates indicated in Table 2 were performed using primers FUJI.EF-F/FUJI.EF-R and TEMP.EF-F/TEMP.EF-R and their corresponding amplification protocols. In the first case, a single fragment of 375 bp was only obtained when genomic DNA was used from either *F. fujikuroi* (Figure 1c), whereas using the second protocol, a fragment of 213 bp was observed when *F. temperatum* DNA was used (Figure 1f).

### 3.2. Study on the Occurrence of Mycotoxins and Mycotoxin-Producing Fungi on Maize Samples

#### 3.2.1. Detection of Mycotoxigenic Aspergillus and Fusarium Species by Specific PCR Assays

All the samples analyzed were positive for amplification using the primer set ITS1/ITS4, indicating the presence of fungal DNA in the samples and its suitability for PCR amplification. The results obtained on the occurrence of mycotoxigenic species, using species-specific PCR assays directly on maize samples at various stages of the production cycle are shown in Table 3. *Aspergillus ochraceus*, *A. westerdijkiae*, *A. steynii*, *F. subglutinans*, *F. temperatum*, *F. equiseti*, *F. culmorum*, *F. sporotrichioides*, *F. poae* and *F. langsethiae* were not detected in the samples using their specific PCR protocols and, therefore, these results are not included in the table. 

It is important to note the presence of at least one mycotoxin-producing species in all the samples taken at the flowering stage. *Aspergillus flavus* was detected at this stage in all samples, at all seasons, and in each of the three batches. The presence of *A. niger* aggregate species (*A. niger* and *A. welwitschiae*) in flowering samples was also confirmed at all times. Contrastingly, the detection of *Fusarium* species was more variable, with three species being detected in 2018, and yet none in 2016.

In the pre-harvest samples, seven mycotoxin-producing species were detected (*F. verticillioides*, *F. proliferatum*, *A. flavus*, *A. parasiticus*, *A. carbonarius*, *A. niger* and *A. welwitschiae*); whereas, during storage in the silo, only five were found (*F. verticillioides*, *F. proliferatum*, *F. graminearum*, *A. flavus* and *A. welwitschiae*).

Regarding the presence of the different fungal species, *A. flavus* was the most frequently detected (89% of samples), followed by the *A. niger* aggregate species (52%). *Aspergillus parasiticus* were only found in a low percentage of pre-harvest samples (11%). On the other hand, *A. carbonarius* was only detected in the flowering phase in 2017 and 2018.

*Fusarium verticillioides* and *F. proliferatum* were the only fumonisin-producing *Fusarium* species that were detected in the maize samples. *F. graminearum* was present in a low percentage of samples (15%), and only in 2018.

Regarding the period of collection, a high number of different mycotoxin-producing species were detected at all crop stages in 2018 compared with the other seasons.

#### 3.2.2. Mycotoxin Contamination

The results regarding mycotoxin analysis on the samples obtained at the moment of harvest or after three months of storage for three consecutive seasons are shown in Table 4. Deoxynivalenol (DON), type A trichothecenes (T-2 and HT-2), zearalenone (ZEA), aflatoxin B_1_ (AFB_1_), and ochratoxin A (OTA) were not detected in any of the samples at any stage.

In the first season of sampling (2016), fumonisins type B (FB) were not detected in the silo, whereas the mean levels of the sum of fumonisins B_1_ and B_2_ (FB_1_ and FB_2_) in the two plots (P1 and P2) studied in pre-harvest were 1068.82 and 661.15 μg/kg, respectively. In no case did the values exceed the regulations for FBs in unprocessed corn, which are established at FB_1_ and FB_2_ > 4000 μg/kg [8]. 

At the second season (2017) FB_1_ and FB_2_ were not detected—neither in the stored grains nor the pre-harvest samples from plot P3. However, FBs were detected at higher levels than in 2016 in pre-harvest samples from the P1 and P2 plots. The FB levels even exceeded the limits established by the European Union in one of the batches from P2.

At the third season (2018), FBs were not detected in the pre-harvest samples from P4, whereas samples from P2 and P3 were contaminated by FBs at low values (mean values 388.04 and 631.07 μg/kg, respectively) in two out of the three batches. During storage, FB_1_ concentration values were near the detection limits of the analysis method and FB_2_ was not detected in any of the silo samples. 

## 4. Discussion

Maize is one of the most important food crops in the world [5]. However, its grains are susceptible to being contaminated by mycotoxin-producing fungi, both in the field and during storage, posing a serious risk to food safety [13]. Several authors have reported the occurrence of mycotoxigenic fungi in maize. *Fusarium verticillioides*, *F. proliferatum* and *F. graminearum* are considered the species that cause the most concern at pre-harvest [12,31], whereas *Aspergillus flavus* causes the most significant concern during the storage of maize [6]. Almost all information regarding the mycotoxigenic potential of these species and their ability to colonize maize comes from in vitro studies. However, the stress on developing maize—particularly during reproductive phases—facilitates infection by the fungi, mycotoxin production and the contamination of the grain [32]. Moreover, it has been reported that, in the field, fungal metabolism changes in order to adapt to unfavorable environmental conditions or limited nutritional availability [33,34]. These authors highlighted the relevance of studies similar to those presented in this manuscript. To date, to our knowledge, there are no studies on the occurrence of mycotoxins and mycotoxin producing-fungi along the complete maize production cycle. The present study is the first one that aims to include all the reproductive cycles and that is focused on the occurrence of the main mycotoxin-producing *Aspergillus* and *Fusarium* species in Spain, from the flowering of the crop to the storage of the grains.

One of the main objectives of this work was to determine the moment when the contamination with potential producers occurs and if it is related with the appearance of the corresponding mycotoxin. Mycotoxigenic fungi can infect maize, even causing ear rots, and afterwards can contaminate grains with mycotoxins [14]. The initial colonization, fungal development, and subsequent mycotoxin production may happen during the cultivation and/or storage of maize [12]. During the three years over which this study was conducted, some *Aspergillus* and *Fusarium* species were detected across the complete production cycle of flowering, harvest and storage. The presence of *A. flavus* was much more frequent and occurred at all stages; in comparison to the three *Fusarium* species detected, which were usually first detected at harvest. It is well-known that *A. flavus* and *F. verticillioides* usually co-occur in corn, since they are able to occupy different niches regarding carbon sources [34]. 

The only toxins detected in this study were fumonisins B_1_ and B_2_ (FB_1_ and FB_2_), the presence of which was consistent at pre-harvest and, in some seasons, reached high levels—although they did not exceed legal limits in silo. Corn is the most susceptible host to contamination, with FUM-producing species often in the field when grains present a high moisture content [35]. In our work, the highest levels of FUMs were observed in pre-harvest in 2017. That season, the weather at harvest time was humid and at high temperatures, with a rainfall of 50 L/m^2^ and a maximum temperature of 42 °C [36], which might have effected an increase in grain moisture which would favor FUM production. 

Several authors proposed different strategies to reduce fungal development as well as mycotoxin production in corn, such as the application of Good Agricultural Practices (GAP), chemical and biological control during cultivation, and proper management during harvest and storage [6,37]. In the farm where this study was carried out, the farmer applied GAP by avoiding stress due to drought or lack of nutrients, rotating crops, using early flowering corn-varieties, and allowing the plots to lie fallow after two or three years of corn cultivation. These measures—that are usually applied—surely should prevent the presence of mycotoxin levels that exceed European Regulations [8]. Removing crop residues, crop rotation, and the practice of fallow seasons are critical factors to prevent fungal colonization and the subsequent mycotoxin production of maize [6,37]. In our study, mycotoxins were not detected in the P3 plot in 2017, nor in P4 in 2018, and both plots lay fallow the preceding season. Furthermore, it is important to note that FBs were detected at the point of harvest in all the other plots that had not lay fallow the previous season—supporting the fact that GAP drastically affect the mycotoxin contamination of the grains. 

In our study, mycotoxin levels in the silos were undetectable in the first two years of the study (2016 and 2017), whereas FB_1_ and FB_2_ were detected at very low levels in 2018. In all seasons, the concentrations found in stored samples were lower than those found at harvest. This might be due to the mixture of different fields in the silo and the grains not corresponding solely to the plots we selected for our study. Maize is one of the most susceptible products to mycotoxin contamination, due to deficient storage conditions, and the extremely low levels observed in this silo might be a reflection of the good agricultural and storage practices engaged in by the farmers. Water activity is one of the main factors affecting mycotoxin growth and production in maize by *A. flavus* and *F. verticillioides,* among others [38]; therefore, the application of this drying process makes the grains less susceptible to fungal colonization and mycotoxin production. In this field, after harvest, the cobs are threshed and homogenized, and the maize grains are subjected to a process of drying which clearly decreased the grain moisture up to levels of 7–8%. These values are in agreement with FAO recommendations to maintain moisture levels less than 15% to prevent fungal growth of fungal species that may be present on fresh grains [39]. Other GAPs that should be applied include the additional control of moisture and temperature during corn storage in silos is crucial to minimizing fungal growth and toxin development [40]. The facilities where this study was conducted were well-ventilated and adequate for the conservation of the corn grains. During the last season of the study, the humidity and temperature data of the silo were recorded across the three months of storage. The relative humidity values were below 65% and the temperature values recorded were always below 10 °C, which are considered poor optimal values for mycotoxin production [41]. 

As mentioned before, detectable levels of FUMs were obtained in the maize grains after three months of storage, though only in 2018. In the same season, *F. graminearum* was also detected in the samples, co-occurring with FUM-producing species. Velluti et al. (2001) described how *F. graminearum* is able to stimulate the growth of *F. verticillioides* when co-occurring, increasing its ability to produce FB_1_ [42]. Therefore, fungal interactions might be also relevant in assessing the mycotoxin risk in maize crops, which supports the importance of this kind of comprehensive study. 

In a climate change scenario, aflatoxins (AFs) have been considered extremely significant as contaminants of maize, mainly in South European countries [13]. However, in our study, AFs were not detected in any samples, although *Aspergillus* section *Flavi* species were detected from the first stages of the production cycle. As mentioned before, AF-production by *Aspergillus* species is significantly affected by humidity and temperature [43]; consequently, maintaining appropriate storage conditions may reduce or even avoid AF contamination. Moreover, several recent studies have reported that many *A. flavus* isolates are unable to synthetize AFs, due to the presence of a non-functional biosynthetic cluster [44,45]. Therefore, it would be interesting to study the capacity of producing AFs of Spanish isolates to uncover the real risk posed by *A. flavus* on maize.

The *A. niger* aggregate species—*A. niger* and *A. welwitschiae*—were consistently detected across all three seasons and each of the steps of production cycle. However, ochratoxin A (OTA) was not found in any case. The characteristics of Spanish isolates of *A. niger* and *A. welwitschiae* have been recently studied and less than 15% are OTA producers [46]. Most of them presented a truncated version of the OTA biosynthetic cluster, that resulted in the loss of their ability to produce the toxin. However, several authors have reported that *A. welwitschiae* and mainly *A. niger* are important FB_2_ producers in maize [46,47]. Therefore, the presence of *A. niger* aggregate species discovered in the present work might be contributing to the FB_2_ levels detected in corn samples.

The rapid and direct identification of the mycotoxigenic species that contaminate maize is a good indicator in predicting the risk of mycotoxin production in the grains during storage, offering an alternative to conventional microbiological procedures in fungal diagnostics. In this context, molecular techniques offer a good alternative tool to detect these species in maize samples [27]. In several published works by our group, species-specific PCR protocols have been developed and successfully applied to detect the main mycotoxin-producing species in cereals [48,49]. 

Previous studies have already optimized specific PCR assays to detect *Fusarium* species that are mycotoxin-producing or pathogens of maize [17,29]. However, the new taxonomic changes and/or the description of new *Fusarium* species of interest in relation to maize [50,51] have supposed that some of these previously described methods gave unspecific results. Therefore, in this work, we have developed and optimized new protocols to detect and identify *F. graminearum*, *F. langsethiae*, *F. fujikuroi*, *F. poae*, *F. sporotrichioides* and *F. temperatum*, using pure DNA or direct maize samples, and we also have modified the PCR conditions in the case of *F. subglutinans* to ensure the specific amplification using the primers described by Scauflaire et al. (2012) [29]. These assays have been tested using the purified DNAs of several isolates of different origins, as well as the DNAs of different, closely related *Fusarium* species frequently associated with corn. The application of these PCR assays directly onto corn grains allows the processing of a high number of samples and reduces the time of analysis compared to conventional methods [27]. In the corn samples analyzed in this work, we were only able to detect one of the species tested using these new optimized protocols—*F. graminearum*—in both flowers and corn grains. However, as mentioned before, the protocols were optimized using the pure DNAs of more than seventeen *Fusarium* species, which guarantee that they may be successful in detecting these species if they are naturally occurring in maize samples.

## 5. Conclusions

Maize is one of the most susceptible cereals to contamination by mycotoxins and mycotoxigenic fungi. In this work, a comprehensive study across the maize production cycle was performed to discover when the contamination by mycotoxigenic fungi appears and its relation to mycotoxin presence in stored maize. The results indicated that *Aspergillus flavus* and fumonisin-producing *Fusarium* species are able to colonize at the earlier stages of the production cycle. However, the application of good agricultural and storage practices by farmers critically minimizes—or even avoids—mycotoxin contamination of maize grains.

## Figures and Tables

**Table 1:** Characteristics of maize samples analyzed in this study, obtained from three growth stages during three consecutive seasons.

Season	Stage	Plot	Variety	Sample Code
2016	ANTHESIS-MALE	P1	DKC 6630	P1.MF-16
		P2	DKC 6442	P2.MF-16
	ANTHESIS-FEMALE	P1	DKC 6630	P1.FF-16
		P2	DKC 6442	P2.FF-16
	PRE-HARVEST	P1	DKC 6630	P1.PRE-16
		P2	DKC 6442	P2.PRE-16
	STORAGE			SILO-16
2017	ANTHESIS-MALE	P1	DKC 6442	P1.MF-17
		P2	DKC 6442	P2.MF-17
		P3	DKC 6630	P3.MF-17
	ANTHESIS-FEMALE	P1	DKC 6442	P1.FF-17
		P2	DKC 6442	P2.FF-17
		P3	DKC 6630	P3.FF-17
	PRE-HARVEST	P1	DKC 6442	P1.PRE-17
		P2	DKC 6442	P2.PRE-17
		P3	DKC 6630	P3.PRE-17
	STORAGE			SILO-17
2018	ANTHESIS-MALE	P2	DKC 6630	P2.MF-18
		P3	DKC 6728	P3.MF-18
		P4	DKC 6442	P4.MF-18
	ANTHESIS-FEMALE	P2	DKC 6630	P2.FF-18
		P3	DKC 6728	P3.FF-18
		P4	DKC 6442	P4.FF-18
	PRE-HARVEST	P2	DKC 6630	P2.PRE-18
		P3	DKC 6728	P3.PRE-18
		P4	DKC 6442	P4.PRE-18
	STORAGE			SILO-18

P (plot); MF (male flower); FF (female flower); PRE (pre-harvest).

**Table 2:** Fungal strains analyzed indicating the origin, species, accession number, and presence (+) or absence (-) of the specific PCR amplification product of *F. temperatum* (FT), *F. langsethiae* (FL) *F. sporotrichioides* (FS), *F. poae* (FP), *F. graminearum* (FG), and *F. fujikuroi* (FJ) using the specific protocols designed in this study.

Strain	Origin	Species	Accession Number	FT	FL	FS	FP	FG	FJ
ITEM 550 ^a^	Poland	*F. sporotrichioides*		-	-	+	-	-	-
ITEM 695 ^a^	USA	*F. sporotrichioides*		-	-	+	-	-	-
ITEM 707 ^a^	Poland	*F. sporotrichioides*		-	-	+	-	-	-
ITEM 1442 ^a^		*F. sporotrichioides*		-	-	+	-	-	-
ITEM 4596 ^a^	Russia	*F. sporotrichioides*		-	-	+	-	-	-
ITEM 4597 ^a^	Russia	*F. sporotrichioides*		-	-	+	-	-	-
CECT 20150 ^b^		*F. sporotrichioides*		-	-	+	-	-	-
CECT 20166 ^b^	Russia	*F. sporotrichioides*		-	-	+	-	-	-
ITEM 6606 ^a^	England	*F. poae*		-	-	-	+	-	-
ITEM 6607 ^a^	England	*F. poae*		-	-	-	+	-	-
MUCL 6114 ^c^	Belgium	*F. poae*		-	-	-	+	-	-
MUCL 7555 ^c^	Belgium	*F. poae*		-	-	-	+	-	-
MUCL 42824 ^c^	Belgium	*F. poae*		-	-	-	+	-	-
CBS 747.97 ^e^	USA	*F. subglutinans*	MN861787	-	-	-	-	-	-
MPE-0990 ^g^		*F. subglutinans*	MN861796	-	-	-	-	-	-
CBS 138.287 ^e^	Mexico	*F. temperatum*	MN861786	+	-	-	-	-	-
F1-VERT	Spain	*F. verticillioides*	MN861741	-	-	-	-	-	-
F3-VERT	Spain	*F. verticillioides*	MN861743	-	-	-	-	-	-
MPA 0999 ^g^	USA	*F. verticillioides*	MN861799	-	-	-	-	-	-
F2-PRO	Spain	*F. proliferatum*	MN861742	-	-	-	-	-	-
F4-PRO	Spain	*F. proliferatum*	MN861745	-	-	-	-	-	-
MPD 4853 ^g^		*F. proliferatum*	MN861797	-	-	-	-	-	-
ITEM 4092 ^a^	Italy	*F. fujikuroi*	MN861805	-	-	-	-	-	+
ITEM 4093 ^a^	Italy	*F. fujikuroi*		-	-	-	-	-	+
ITEM 4094 ^a^	Italy	*F. fujikuroi*		-	-	-	-	-	+
ITEM 4095 ^a^	Italy	*F. fujikuroi*		-	-	-	-	-	+
MPC1993 ^g^		*F. fujikuroi*		-	-	-	-	-	+
MPC1995 ^g^	Taiwan	*F. fujikuroi*		-	-	-	-	-	+
PRC 14a ^d^		*F. fujikuroi*		-	-	-	-	-	+
PRC 19a ^d^		*F. fujikuroi*		-	-	-	-	-	+
ITEM 6013 ^a^	USA	*F. globosum*	MN861806	-	-	-	-	-	-
ITEM 1590 ^a^	Italy	*F. globosum*	MN861808	-	-	-	-	-	-
F23-LANG	Spain	*F. langsethiae*	MN861761	-	+	-	-	-	-
L.3.1	UK	*F. langsethiae*		-	+	-	-	-	-
L.3.2	UK	*F. langsethiae*		-	+	-	-	-	-
NRRL 28585 ^f^	Venezuela	*F. graminearum*		-	-	-	-	+	-
NRRL 28436 ^f^	France	*F. graminearum*		-	-	-	-	+	-
NRRL 29020 ^f^	South Africa	*F. graminearum*		-	-	-	-	+	-
NRRL 29148 ^f^	USA	*F. graminearum*		-	-	-	-	+	-
NRRL 26755 ^f^	South Africa	*F. graminearum*		-	-	-	-	+	-
NRRL 13818 ^f^	Japan	*F. graminearum*		-	-	-	-	+	-
NRRL 29169 ^f^	USA	*F. graminearum*		-	-	-	-	+	-
NRRL 28585 ^f^	New Zealand	*F. graminearum*		-	-	-	-	+	-
ITEM 628 ^a^	Yugoslavia	*F. culmorum*		-	-	-	-	-	-
ITEM 4335 ^a^		*F. culmorum*		-	-	-	-	-	-
ITEM 6717 ^a^	Hungary	*F. culmorum*		-	-	-	-	-	-
ITEM 6718 ^a^	Hungary	*F. culmorum*		-	-	-	-	-	-
Be1:H3-1/1G	Spain	*F. avenaceum*		-	-	-	-	-	-
ITEM 4094 ^a^		*F. thapsinum*	MN861807	-	-	-	-	-	-
MPB 3852 ^g^		*F. sacchari*	MN861798	-	-	-	-	-	-
VI01093	Norway	*F. equiseti*		-	-	-	-	-	-
F100	Spain	*F. oxisporum*	MN861795	-	-	-	-	-	-
F103	Spain	*F. solani*	MN861794	-	-	-	-	-	-

Strains retrieved from ^a^ Agrofood Microbial Culture Collection (ISPA, Bari, Italy), ^b^ Spanish Type Culture Collection (CECT, Valencia, Spain), ^c^ Belgian Coordinated Collections of Micro-organisms (BCCM, Brussels, Belgium), ^d^ Hurbarium Collections at the Charles University (PRC, Prague, Czechia), ^e^ Westerdijk Fungal Biodiversity Institute Collection (CBS-KNAW, Utrecht, The Netherlands), ^f^ Agricultural Research Center Culture Collection (USDA, Washington, DC, USA). ^g^ Strain from *G. fujikuroi* mating populations A-H.

**Table 3:** PCR detection of *Fusarium* and *Aspergillus* species in pre-harvest and stored maize samples using species-specific assays. The presence (+) or absence (-) of the specific PCR amplification product is indicated for each replicate (1, 2, 3).

	*F. verticillioides*			*F. proliferatum*			*F. gramunearum*			*A. flavus*			*A. parasiticus*			*A. carbonarius*			*A. niger*			*A. welwitschiae*		
**SAMPLES**	1	2	3	1	2	3	1	2	3	1	2	3	1	2	3	1	2	3	1	2	3	1	2	3
P1.FM-16	-	-	-	-	-	-	-	-	-	+	+	+	-	-	-	-	-	-	-	-	-	-	-	-
P2.FM-16	-	-	-	-	-	-	-	-	-	+	+	+	-	-	-	-	-	-	-	-	-	-	-	-
P1.FF-16	-	-	-	-	-	-	-	-	-	+	+	+	-	-	-	-	-	-	-	-	-	-	-	-
P2.FF-16	-	-	-	-	-	-	-	-	-	+	+	+	-	-	-	-	-	-	-	-	-	-	-	-
P1.PRE-16	+	+	+	-	-	-	-	-	-	+	-	-	-	-	-	-	-	-	-	-	-	+	+	+
P2.PRE-16	+	+	+	+	+	+	-	-	-	+	+	+	-	-	-	-	-	-	+	-	-	+	+	+
SILO-16	+	+	+	+	+	+	-	-	-	+	+	+	-	-	-	-	-	-	-	-	-	-	-	-
P1.FM-17	-	-	-	-	-	-	-	-	-	+	+	+	-	-	-	-	-	-	+	+	+	+	-	-
P2.FM-17	-	-	-	-	-	-	-	-	-	+	+	+	-	-	-	+	-	-	-	-	-	-	-	-
P3.FM-17	+	-	-	-	-	-	-	-	-	+	+	+	-	-	-	-	-	-	-	-	-	-	-	-
P1.FF-17	+	+	+	+	-	-	-	-	-	+	+	+	-	-	-	+	+	+	+	+	+	+	-	-
P2.FF-17	-	-	-	-	-	-	-	-	-	+	-	-	-	-	-	-	-	-	+	-	-	-	-	-
P3.FF-17	+	-	-	-	-	-	-	-	-	+	+	+	-	-	-	-	-	-	+	+	+	+	+	+
P1.PRE-17	-	-	-	-	-	-	-	-	-	+	+	+	+	+	-	-	-	-	+	+	+	+	+	+
P2.PRE-17	+	+	+	+	+	+	-	-	-	+	+	+	-	-	-	-	-	-	-	-	-	-	-	-
P3.PRE-17	+	-	-	+	-	-	-	-	-	+	-	-	+	-	-	-	-	-	+	+	+	+	-	+
SILO-17	+	+	+	+	-	-	-	-	-	+	+	+	-	-	-	-	-	-	-	-	-	-	-	-
P2.FM-18	+	+	+	-	-	-	+	+	+	+	+	+	-	-	-	-	-	-	-	-	-	+	+	+
P3.FM-18	+	+	+	+	+	+	+	+	-	+	+	+	-	-	-	+	+	+	-	-	-	+	+	+
P4.FM-18	-	-	-	+	-	-	+	+	+	+	+	+	-	-	-	+	+	+	+	+	+	+	+	+
P2.FF-18	+	+	+	+	+	+	-	-	-	+	+	+	-	-	-	-	-	-	-	-	-	-	-	-
P3.FF-18	+	+	+	-	-	-	-	-	-	+	+	+	-	-	-	-	-	-	-	-	-	+	-	-
P4.FF-18	-	-	-	-	-	-	-	-	-	+	+	+	-	-	-	+	-	-	-	-	-	+	+	+
P2.PRE-18	+	+	+	+	+	+	-	-	-	+	+	-	-	-	-	-	-	-	-	-	-	-	-	-
P3.PRE-18	+	+	+	+	+	+	-	-	-	-	-	-	-	-	-	-	-	-	-	-	-	-	-	-
P4.PRE-18	+	+	+	-	-	-	-	-	-	-	-	-	+	+	-	-	-	-	-	-	-	-	-	-
SILO-18	+	+	+	+	+	+	+	+	+	+	+	+	-	-	-	-	-	-	-	-	-	+	+	+

PCR, polymerase chain reaction; (+) positive amplification and (-) no amplification. P (plot); MF (male flower); FF (female flower); PRE (pre-harvest).

**Table 4:** Fumonisin concentrations (FB_1_ and FB_2_) in the pre-harvest (PRE) and stored maize samples, across three seasons. Each value corresponds to the mean of two replicates ± standard error. The detection limits were 180 and 60 μg/kg in the cases of FB_1_ and FB_2_, respectively.

Season	Plot	Samples	FB_1_ (μg/kg)	FB_2_ (μg/kg)
2016	P1	PRE 1.1-16	1219.44 ± 2.21	214.86 ± 1.96
		PRE 1.2-16	1289.89 ± 14.75	218.15 ± 5.82
		PRE 1.3-16	204.14 ± 0.62	ND
	P2	PRE 2.1-16	ND	ND
		PRE 2.2-16	ND	ND
		PRE 2.3-16	1334.74 ± 31.57	168.71 ± 2.62
		SILO-16	ND	ND
		SILO-16	ND	ND
		SILO-16	ND	ND
2017	P1	PRE 1.1-17	ND	ND
		PRE 1.2-17	3658.78 ± 75.79	740.06 ± 4.08
		PRE 1.3-17	ND	ND
	P2	PRE 2.1-17	5902.87 ± 45.73	606.41 ± 29.06
		PRE 2.2-17	ND	ND
		PRE 2.3-17	1496.14 ± 33.05	312.32 ± 2.02
	P3	PRE 3.1-17	ND	ND
		PRE 3.2-17	ND	ND
		PRE 3.3-17	ND	ND
		SILO-17	ND	ND
		SILO-17	ND	ND
		SILO-17	ND	ND
2018	P2	PRE 2.1-18	371.53 ± 0.17	111.24 ± 3.24
		PRE 2.2-18	355.78 ± 6.83	85.86 ± 1.62
		PRE 2.3-18	ND	ND
	P3	PRE 3.1-18	870.63 ± 9.98	215.46 ± 5.22
		PRE 3.2-18	455.35 ± 6.30	111.78 ± 0.18
		PRE 3.3-18	ND	ND
	P4	PRE 4.1-18	ND	ND
		PRE 4.2-18	ND	ND
		PRE 4.3-18	ND	ND
		SILO-18	180.25 ± 1.40	ND
		SILO-18	198.98 ± 0.53	ND
		SILO-18	186.03 ± 0.88	ND

ND: non detected; P (plot); PRE (pre-harvest).
